# Concerns of Parental Substance Abuse and Mental Health Problems Reported to Child Welfare Services—Testing a Moderated Mediation Model for Paths From Reports to Substantiated Concern and Service Provision

**DOI:** 10.3389/fpsyt.2022.781332

**Published:** 2022-02-28

**Authors:** Svein Arild Vis, Camilla Lauritzen, Karen J. S. Havnen, Charlotte Reedtz, Bjørn Helge Handegård

**Affiliations:** ^1^Regional Center for Child and Youth Mental Health and Child Welfare, UiT-The Arctic University of Norway, Tromsø, Norway; ^2^Regional Center for Child and Youth Mental Health and Child Welfare, NORCE Norwegian Research Centre, Bergen, Norway

**Keywords:** child protection, investigation, assessment, parental mental health, parental substance abuse, decision making, substantiation, service provision

## Abstract

**Background:**

Parental mental health and substance abuse problems are found in reports of concern to child protection and welfare services. The aim of this study was first to investigate what characterized these reports and how they differed from reports with other types of concerns. Two hypotheses were tested. The first hypothesis was (i) if a report contains concerns about mental health and substance abuse problems, the likelihood of service provision was mediated by substantiation status. The second hypothesis was (ii) that the threshold for substantiation of such problems differed depending on child age, single parent status, and the presence of other child and parent related problems.

**Method:**

The study was designed as a case file study which was carried out retrospectively (*N* = 883). A conceptual model was tested in two steps. First a mediation model with direct and indirect paths from reports of concerns through substantiation decision to service provision was tested. Then a second model was expanded to also include moderators for the indirect effects of reported concerns on substantiation decisions.

**Results:**

A total of 33.1% of reports about substance abuse and 41.7% of reports about parental mental illness concerns were provided services. The first hypothesis was confirmed. There is a negative direct effect and a positive mediated effect of reported concern on service provision. The second hypothesis was not confirmed. We failed to identify any significant moderating effect of child age, single caregiver status, or number of child problems, upon the threshold for substantiation of mental health and drug abuse problems.

**Conclusions:**

The total effect of reports about mental illness and substance abuse upon service provision was low. Service provision in cases with suspected substance abuse and/or mental illness is highly dependent upon substantiation of that specific problem. Substantiation threshold is not impacted by other case characteristics. This is surprising because there are good theoretical reasons to assume that parental drug abuse and or mental illness are potentially more detrimental to child health, development and safety if the child is younger, if the parent is a single caregiver, and there are many other parallel concerns.

## Introduction

A public health approach to prevent adverse outcomes and improve quality of life for children when there is a concern about the parent's mental health or substance use is important. The Norwegian Child Welfare and Protection Services (CWPS) are obligated to ensure that children and youth who live in conditions that may be detrimental to their health and development receive necessary support and care. A substantial proportion of the reports of concern the CWPS receives is about parental mental health problems (12.0%) and parental substance abuse (16.9%) (1). There is little knowledge about whether these circumstances lead to reports of concern being substantiated. Substantiation implies that a case is considered by the CWPS to cause a serious concern for the child's health and development. When a case is substantiated, the family will normally be offered some form of voluntary support service by the CWPS. Usually this takes the form of parental guidance and counseling conducted by a social worker. This may be in addition to, but usually not as a substitute for other health care or social services.

It should be an aim for health care and social services to work together to identify and provide services for children and their parents in families struggling with parental mental health problems and/or substance abuse. Little is known about how factors such as child age, parental custody, and the presence of other risk factors influence the judgement and risk assessments of social workers within the CWPS. It is important to learn more about this because early identification and service provision may help prevention of trans-generational transmission of mental health problems. When the problems influence the child's everyday life and functioning the CWPS can offer supportive interventions for these families. However, this depends on the problems being identified and reported by other services or individuals.

One of the major challenges in mental health care and other health services has been to identify children of parents with a mental illness (COPMI) (2). According to the Norwegian Child Welfare Act of 1992, professionals in institutions bound by the professional duty of confidentiality (i.e., teachers, day care personnel, health care services) are required to report cases of concern to the CWPS. Adult psychiatric services have started to recognize the parenting challenges patients have, and to acknowledge the need for interventions to support parenting and the patients minor children (3). One approach to increase identification of COPMI and increase provision of services in Norway has been to appoint designated child responsible personnel in adult mental health services. The role of the child responsible personnel has been to record information about patients' children and establish collaboration with services that could provide patients and their families with support (2). We are therefore interested in looking more closely at what proportion of cases reported to the CWPS originate from health care services.

Parents struggling with mental health issues may potentially experience the development of a variety of problems in their offspring. These children run a higher risk of abuse and neglect, depression, anxiety disorders, substance abuse, eating disorders, conduct problems, and academic failure (4–6). The ongoing spiral, where mental disorders are being transmitted from one generation to the next, is one of the core mechanisms in the development of mental illness (3). Previous research found that one in five minors have a parent with mental illness (7), and that between 44 and 74% of these children develop psychosocial or mental health problems (8).

In the same way parental substance abuse (PSA) is a well-established risk factor for a variety of negative psychosocial outcomes for children (9–14), but some studies have also focused on “resilient” children of substance abusers, like the “classical” study by Werner (15). American studies have widely documented the association between PSA and child maltreatment, and both American and British studies have recognized PSA as a major concern for the CWPS (16–18).

Early parent-child interactions have shown to be important in child development (19). A focus on how parental substance abuse and mental health problems influence the interaction between parents and their children is crucial for the CWPS in order to determine risk for the child's health and development. Being a single parent or caregiver leaves the child more dependent on one caregiver because there is no one else to compensate for parent-child interaction problems. Hence, single caregiver status is considered a strong risk factor for child developmental problems in such cases (20). Additionally, the potential risk is larger for very young children compared to older children. One of the reasons for this is that young children, i.e., under school age are more dependent upon their parents for provision of basic care and safety. Whereas, parental mental health problems also affect the emotional support and guidance for older children, deficiencies in parenting due to mental health problems also poses a potential safety risk when young children are involved.

Children and adolescents in contact with the CWPS have been found to have an elevated risk for mental health problems. In a large Norwegian study, Iversen et al. (21) found that 56% of children receiving in-home services from the CWPS had mental health problems, compared to 8% among those who did not. Additionally, several Norwegian studies (22, 23) and international studies (24–26) have documented that children in out-of-home care have mental health problems or disorders to a significantly larger extent than the general population of minors. In a recent study in Norway, researchers found that youth in foster care had lower scores on life quality compared to a general sample of adolescents, and that the life quality among those in foster care was comparable to COPMI (27).

In some contrast to the above mentioned studies Havnen et al. (28) found that Norwegian children placed in out-of-home care due to parental substance abuse had less mental health problems and better prosocial behavior than children placed due to other reasons. Research on mental health issues of children with PSA problems in the CWPS is however scarce, and the findings are somewhat divergent (26, 29). Findings about sociodemographic characteristics of the PSA children, are more consistent, as several studies have reported that children placed out-of-home because of PSA were younger, more often girls and more often had single caregivers than other children placed out-of-home (16, 18, 28, 29). In a study of COPMI conducted by Reedtz et al. (30), similar characteristics were discovered. The study assessed the circumstances and characteristics of COPMI when a parent was receiving treatment in the adult mental health services. Two thirds of the children were aged 0–11, and a large proportion had single caregivers (30).

A recent Norwegian study of the CWPS (31) compared problems identified in reported concerns to the services with concerns described by the CWPS in their concluding investigation reports. Reports about abuse/neglect were substantiated in about half the cases and rarely identified as new problems, while concerns about children's functioning, parenting competencies and parent-child interactions were often assessed as worrying, also in cases where this was not reported initially. Reports about parental substance abuse were substantiated in <50% of the cases and seldom detected as a new problem, whereas parental mental health problems were more likely to be detected as new problems irrespective of the content in the initial report of concern. The systematic change in problem profile from initial reports of concern to the concluding assessment reports from the CWPS, led the authors to suggest that the assessment process during the investigation is influenced by a tendency of CWPS workers to identify certain risk factors more than others (31). A seemingly predominant focus on parent-child interactions and a lack of suitable interventions that target many of the possible risk factors, might hinder CWPS workers from detecting problems related to parental mental health, as well as parental substance abuse. If this is the case, it may represent an erroneous basis for assessment conclusions made by the CWPS, as these problems represent serious risk factors in child development.

To understand the context of our study it is important to note that the Norwegian CWPS differs in some ways from the British and the US CPS (32). Whereas, the British and US services traditionally have been described as directed primarily toward protecting children against neglect and abuse, the Nordic CWPS are described as more directed toward supporting children and families in need, in order to prevent out-of-home placements (33–35). Although the development toward prevention and home-based support through differential response systems has been seen in the US and other countries (36) there is still a disproportionate number of children of PSA being placed into out-of-home care. In the US the proportion of PSA children in out-of-home care increased from 18.5% in the year 2000 to 38.9% in 2019. There were however substantial differences between states (37). Results from a Canadian longitudinal study on placement risk (38) show that for younger children below the age of nine, increased placement risk is explained by family difficulties whereas increased placement risk for older children is explained by behavioral problems. There are currently no epidemiological studies linking PSA or parental mental health problems to risks for out-of-home care in Norway. The main activity of the Norwegian CWPS, consists of voluntary consent-based interventions (71%), while relatively few of the families receiving services (29%) are placed out-of-home by court orders (1). In line with the public welfare tradition, the threshold to report cases of concern to the Norwegian CWPS is low. About 80% of all reports of concern to the CWPS in Norway are screened-in for further investigation. Of these, only 40% of the investigations are concluded with service provision (31, 39). The most common reason for service provision from the CWPS is parenting problems (23%), while parental mental health problems and substance abuse only account for 8 and 5%, respectively (1).

The current study is based upon the theory of the General Assessment and Decision-Making model in child protection (40). The theory states that the assessment dimension of risk, i.e., the level of concern in a CWPS investigation, is dependent upon the case factors influencing the assessment and the threshold for taking action. If the evaluation of case factors indicate that the weight and amount of evidence is large enough to raise an alarming concern for the child, then the case is substantiated, and services will be offered to the family. The threshold level for substantiation is however not defined by an objective proxy but is rather influenced by the views, experience, and knowledge of the decision-maker. The actions of social workers are in turn influenced by organizational factors, such as the routines and regulations within the CWPS agency, as well as factors external to CWPS, e.g., the capacity and service provision by other health care services. This study is focusing on the interaction of different case factors and how these influence the threshold levels determined by social workers when concluding an investigation of parental mental health problems or substance abuse.

## Aims and Hypotheses

The purpose of the current study is to investigate (a) who reports concerns about parental substance abuse or mental health problems and what are the characteristics of these reports of concern, (b) what are the direct and mediated effects of reports about COPMI and PSA upon service provision, and (c) what are the thresholds for substantiation of parental mental health problems and parental substance abuse moderated by other case characteristics.

We hypothesize that service provision is dependent upon the CWPS's investigation and assessment of the case. Furthermore, we believe the threshold for substantiation of concerns as well as provision of services is lower the younger the child is, in cases where parents have substance abuse problems or mental health problems and are single caregivers. We also hypothesize that in cases where the reported substance abuse problems and mental health problems coinside with other child problems and parenting problems, there is a lower threshold for substantiation and service provision.

## Methods

The study was designed as a case file study which was carried out retrospectively. A total of 1,365 cases were randomly drawn from all referrals registered in the 16 participating agencies in the period of January 2015 to June 2017. The agencies represented (i) six districts from the three major cities in Norway with a population ranging from 190,000 to 680,000, (ii) six regional cities with a population ranging from 20,000 to 80,000, and (iii) four agencies from smaller towns and rural areas with a population below 15,000. The number of cases from each agency varied between 50 and 150 depending on the size of the agency. The reason why we sampled agencies by size is that we wanted the number of cases drawn from each agency to be approximately the same proportion of the total available sample from that agency.

Data was collected and coded from case records. A data entry form was developed and tested for interrater reliability by independent coding of 20 cases by two researchers. The results showed an average interrater agreement of 86.9%. A total of 13 variables had low reliability (<80% interrater agreement). Three of those were eliminated from the form due to the conclusion that reliable information could not be obtained. The remaining 10 variables were reformulated, and the coding manual was revised with better explanation of codes. After this revision the reliability of the instrument was re-tested by independent coding of 42 cases by two researchers. At this second step, average interrater agreement was 90.8%. In health research, an interrater agreement over 80% is generally considered acceptable (41). The variables and the codes from the form are available from the corresponding author upon request.

### Participants

For the present analysis we included all the cases that were subject of a child protection investigation, and which were concluded in an investigation report (*n* = 883). We did not include cases that were screened out without any further investigation (*n* = 242) and cases that did not have a concluding report (*n* = 240). The reason for this is that we were not able to determine whether or not the reported concern had been substantiated in those cases.

There were 54.0% boys (*n* = 477) in the sample and the mean age was 9.0 years (SD = 5.0). In a total of 40.8% of the referrals, the family had immigrant background. Immigrant background was defined as the child or one of the parents being born in a country other than Norway.

The sample was representative for the population of cases involved with the CWPS in Norway with respect to child age, child gender, and the proportion of cases screened out or screened in for service provision.

### Measures

The dependent variables investigated in this article are whether parental mental illness or substance abuse was substantiated and if services were provided. Predictors were (i) child age (ii) caregivers' civil status, (iii) number of substantiated concerns about child development and health, (iv) number of substantiated concerns about parenting problems.

Possible substantiated child development and health related concerns were age-adequate development, mental health problems, child crime/substance abuse, externalizing behavior problems, functioning in school/kindergarten, emotional problems, social problems with peers, social problems with adults or conflict with adults. Each problem was counted as substantiated or not.

Possible substantiated concerns about parenting problems were deficiencies in parental stimulation/guidance/boundary setting, basic care for the child, parents' emotional availability, or parents' protection of the child.

Summary of coding of included variables and their possible values are shown in Table 1.

**Table 1:** Included variables and their coding.

**Data source**	**Variables**	**Values**
Reports of concern	Reported parental substance abuse problem	0 = no 1 = yes
	Reported parental mental illness	0 = no 1 = yes
	Single caregiver	0 = no 1 = yes
	Child age	0–17
Investigation report	Number of child problems substantiated	0–9
	Number of parenting problems substantiated	0–4
	Substance abuse substantiated	0 = not substantiated 1 = substantiated
	Mental health problems substantiated	0 = not substantiated 1 = substantiated
CPS conclusion	Service provision	0 = no 1 = yes

### Statistical Analyses

**Figure 1:**
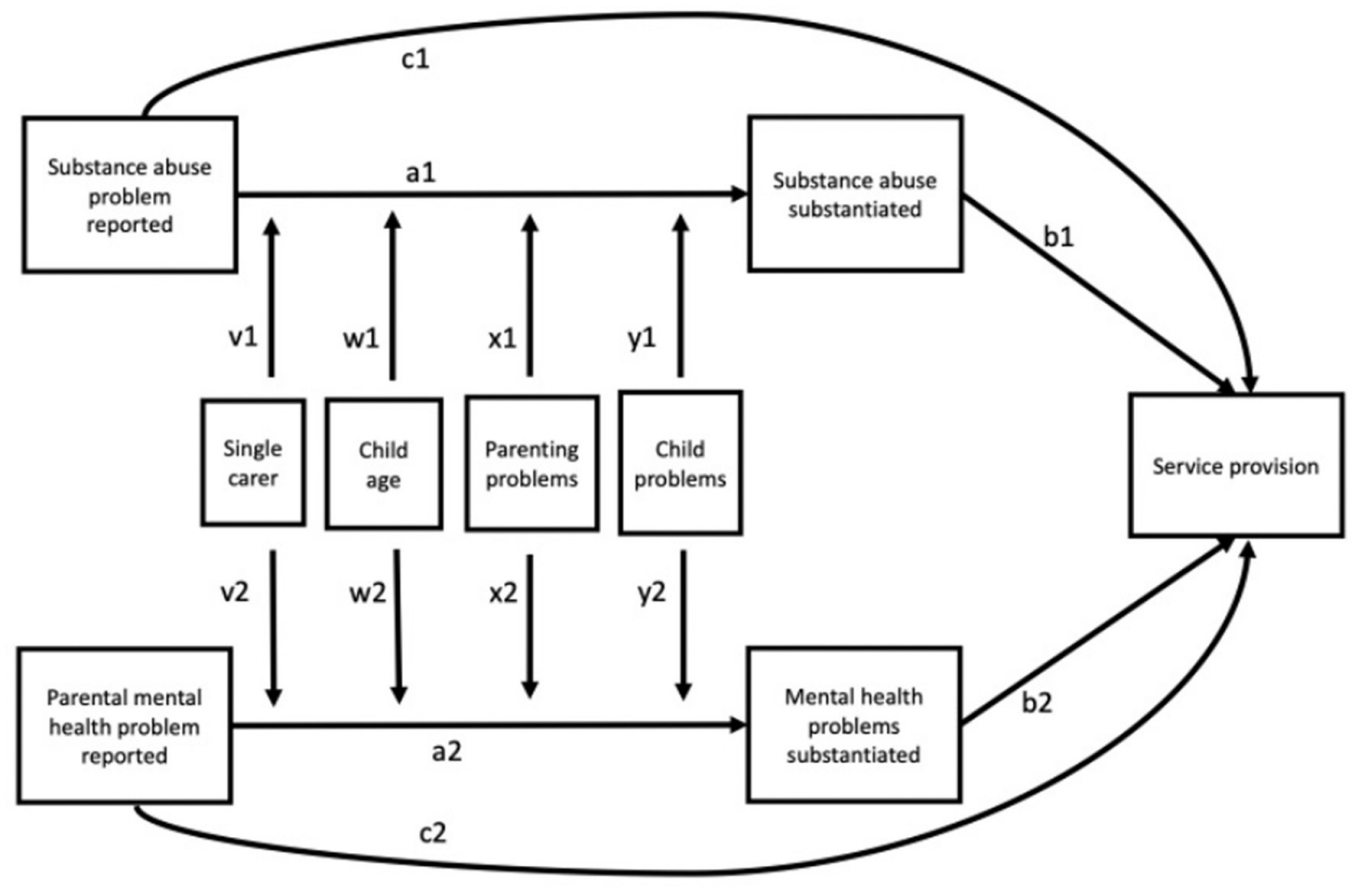
The conceptual model. In the moderation analysis, the substance abuse paths (a1, b1, c1) and the parental mental health paths (a2, b2, c2) were tested separately.

Analysis was carried out in Mplus. The conceptual model shown in Figure 1 was tested in two steps. First the mediation model (a, b, and c paths) was tested for each of the paths from reported mental illness or substance abuse to service provision. Then a second model was expanded to also include the moderators (Figure 1). When programming the model in Mplus we followed the recommendations by Stride et al. (42) to test indirect and total effects of each possible combination of high, medium and low values for the moderators using the model constraint function. For the age variable, low was set to 4 years, medium was 9 years and high was 14 years. For the variables: number of parenting problems and number of child problems, low was set to zero, medium was one and high was three.

We used bootstrapping and the weighted least square mean and variance adjusted estimations.

Chi-square tests were used to test differences in distribution of reporters for cases with mental health problems/substance abuse problems and reporters in cases without mental health problems/substance abuse problems.

## Results

Cases with concerns about parental mental illness and substance abuse problems are not surprisingly often reported by healthcare services. It is worth noting however that private parties, such as a relative, a friend or a neighbor seem to be much more involved in reporting those kinds of concerns compared to the CWPS reports in general. This is particularly true for reports concerning substance abuse problems where about one third of the reported concerns originated from a non-mandated reporter. For the overall reports of concerns to CWPS in Norway however, about four out of five reported concerns were submitted by a professional (Table 2).

**Table 2:** Reporters of cases with concerns about parental mental health problems or substance abuse to CPS.

	**Reports with parental mental health problems (*n* = 132)** ***N* (%)**	**Reports with parental substance abuse problems (*n* = 160)** ***N* (%)**	**Total sample (*n* = 883)** ***N* (%)**
Health care services	50 (37.9)	40 (25.0)	175 (19.8)
School	11 (8.3)	9 (5.6)	188 (21.3)
Police	12 (9.1)	28 (17.5)	127 (14.4)
Social services	10 (7.6)	5 (3.1)	53 (6.0)
CPS agency	14 (10.6)	25 (15.6)	132 (14.9)
Private parties	32 (24.2)	48 (30.0)	187 (21.2)
Other	3 (2.3)	5 (3.1)	21 (2.4)

*The overall test for differences in who referred cases with mental illness [$\chi_{\left(6\right)}^{2}$ = 44.1] and substance abuse [$\chi_{\left(6\right)}^{2}$ = 36.8] compared to other cases were both highly significant (p < 0.001). The tests are comparisons of the distribution of reporters for cases with mental health problems/substance abuse problems and reporters in cases without mental health problems/substance abuse problems*.

There are also other differences between concerns about parental mental illness and substance abuse compared to the total amount of reported concerns (Table 3). First, these reports were related to children who were about 1 year younger compared to the other reports of concern, and their caretakers were more often a single parent. There were some differences between reports of concerns regarding parental mental illness and those with concerns about substance abuse, with respect to what other problems were substantiated because of the investigation. When the report was about substance abuse, problems related to child development and heath were less frequently identified. When the reported concern was about parental mental illness more parenting problems were identified. The reported concerns about mental illness and substance abuse problems were substantiated in about 50–60% of the cases. Substantiation of a problem does however not always lead to provision of services for the family. Services were provided in about 38% of the cases and was not more or less likely in cases about mental illness and substance abuse than in other types of cases.

**Table 3:** Characteristics of reports with suspected parental mental illness or substance abuse compared to the total sample.

	**Total Sample** **(*n* = 883)** ***M* (SD)**	**Reports with alleged parental mental health problems** **(*n* = 132)** ***M* (SD)**	**Reports with alleged parental substance abuse** **(*n* = 160)** ***M* (SD)**
Child age	9.0 (5.0)	8.0 (5.4)^*^	7.7 (5.1)^**^
Number of substantiated child related problems	1.1 (1.7)	0.9 (1.6)	0.6 (1.4)^***^
Number of substantiated parenting problems	0.6 (0.9)	0.8 (1.1)^*^	0.5 (1.0)
	***N*** **(%)**		
Single care	299 (33.9)	56 (42.4)^*^	69 (43.1)^**^
Substantiated mental health problem	144 (16.3)	77 (58.3)^***^	43 (26.9)^***^
Substantiated drug abuse problem	110 (12.5)	24 (18.2)^*^	86 (53.8)^***^
Service provision	339 (38.4)	55 (41.7)	53 (33.1)

*Significance levels are*

* *p < 0.05*;

** *p < 0.01; and*

*** *p < 0.001. Testes are t-tests and chi-square tests for differences between cases where the referral concern is present vs. all other cases*.

**Figure 2:**
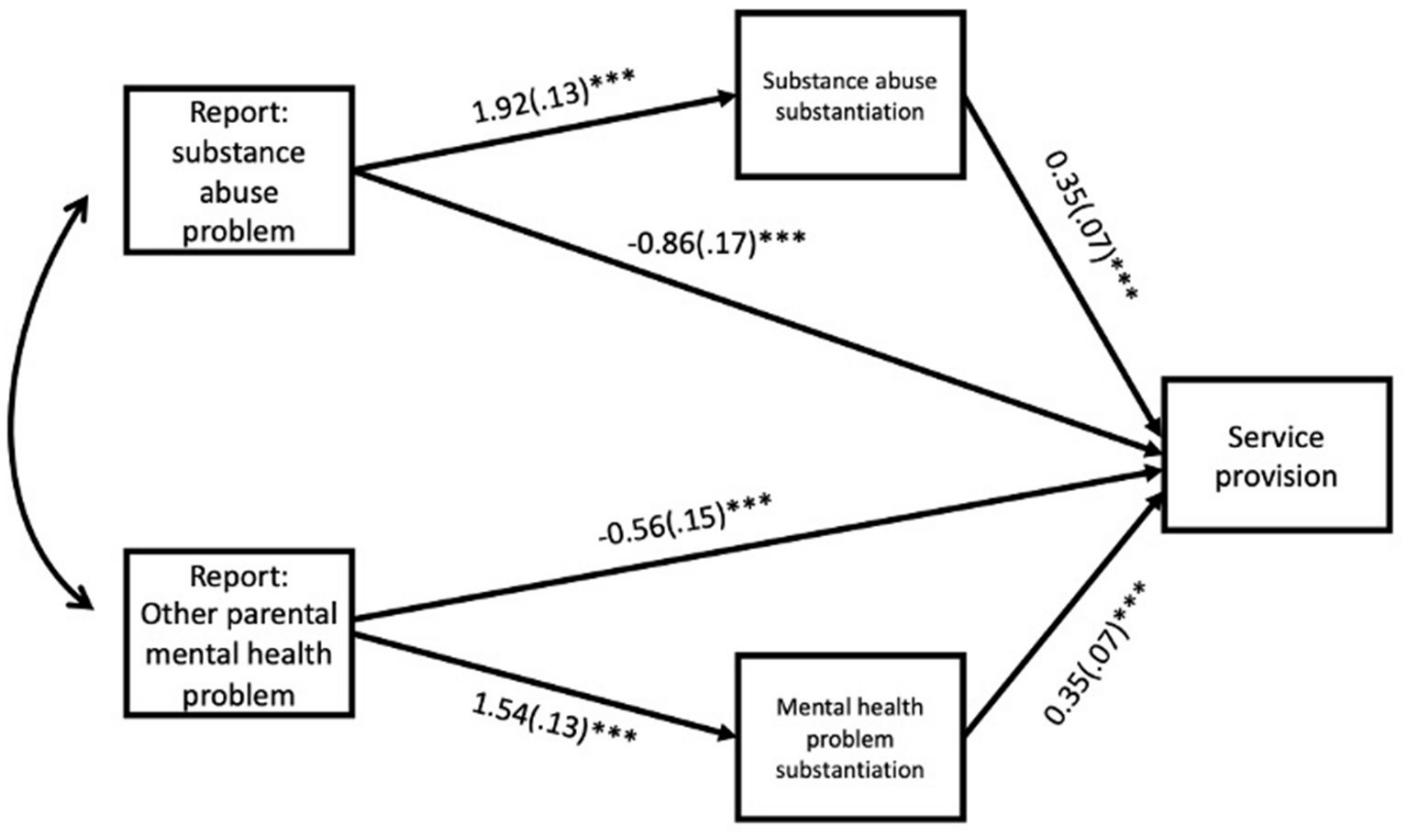
Direct and indirect effects from referral concern to service provision. ***Indicate *p* < 0.001. The specific indirect effect from reported substance abuse problem to service provision was 0.67 (0.11)***. The specific indirect effect from reported parental mental health problem to service provision was 0.67 (0.14)***.

The results from the mediation analysis showed, as should be expected, that the total effects of the reported concerns about parental mental illness (OR = 1.22, *p* = 0.33) or substance abuse (OR = 0.72, *p* = 0.10) upon service provision is low and non-significant. As shown in Figure 2, the direct effects are negative, and the indirect effects are positive. This indicates that service provision in these types of reports is highly dependent upon whether or not that specific problem is substantiated by CWPS. It is also worth noting that there were strong effects from reported concerns to substantiation decision. This means that substantiation of parental substance abuse or mental health problems is highly dependent upon being identified as a concern in the report to the CWPS, substance abuse problems more so than other mental health problems. The total odds ratio for reports of mental health problems to service provision via substantiation was OR = 1.86 (95% CI: 1.40–2.33) and the odds ratio from substance abuse reports to service provision through substantiation was OR = 1.81 (95% CI: 1.23–2.39). The path effects for the mediation model are shown in Figure 2. The log scale estimates for indirect effects are found in the table's two sub-notes.

The analysis of moderated paths from reports of concern to substantiation did not identify any statistically significant moderators, i.e., there were no statistically significant interaction effects between reported concerns and child age, single care status, or the number of substantiated child or parenting problems, upon substantiation decision for either mental health problems or substance abuse. The regression estimates for the moderated mediation models are shown in Tables 4, 5.

**Table 4:** Regression estimates for the moderated mediation paths from referrals of parental mental health problems to service provision (*n* = 883).

**Dependent variable**	**Predictor**	**WLSM estimate**	**Standard error**	***P***
Service provision				
	Constant	0.629	0.118	<0.001
	Referral for mental illness (c2 path)	−0.537	0.351	0.126
	Substantiated mental illness (b2 path)	0.534	0.065	<0.001
Substantiated mental illness				
	Constant	1.476	0.165	<0.001
	Referral for mental illness	1.459	0.303	<0.001
	Child age	−0.031	0.014	0.022
	Singelcare = yes	0.142	0.127	0.265
	Substantiated child problems	0.164	0.036	<0.001
	Substantiated parenting problems	0.463	0.070	<0.001
	Referral by age (w2 path)	0.002	0.029	0.933
	Referral by single care (v2 path)	−0.410	0.280	0.143
	Referral by child problems (y2 path)	−0.068	0.095	0.475
	Referral by parenting problems (x2 path)	0.322	0.173	0.063

**Table 5:** Regression estimates for the moderated mediation paths from referrals of parental substance abuse problems to service provision (*n* = 883).

**Dependent variable**	**Predictor**	**WLSM estimate**	**Standard error**	***P***
Service provision				
	Constant	0.576	0.122	<0.001
	Referral for substance abuse (c1 path)	−1.177	0.328	<0.001
	Substantiated substance abuse (b1 path)	0.553	0.082	<0.001
Substantiated substance abuse				
	Constant	2.267	0.282	<0.001
	Referral for substance abuse	2.101	0.362	<0.001
	Child age	−0.009	0.016	0.575
	Single care = yes	0.002	0.156	0.988
	Substantiated child problems	0.159	0.036	<0.001
	Substantiated parenting problems	0.672	0.076	<0.001
	Referral by age (w1 path)	−0.013	0.029	0.633
	Referral by single care (v1 path)	−0.042	0.286	0.885
	Referral by child problems (y1 path)	0.049	0.168	0.771
	Referral by parenting problems (x1 path)	0.014	0.184	0.939

*Path name refers to Figure 1*.

Since there were no significant moderating effects of child age, single care status or the number of substantiated child or parenting problems on the association between reported concerns and substantiation decision, conditional indirect effects are not reported. Thus, we have not sufficient evidence to claim that the indirect effects vary depending on the age of the child, single care status, or the number of substantiated child or parenting problems. The highest indirect effect for reported concerns about substance abuse with moderators present, were in cases where the parent was not a single caregiver, the child age was young (4 years) and there were three other substantiated child and parenting concerns (1.235, S.E = 0.416, *p* = 0.003). The lowest indirect effect with moderators present were when there was a single caregiver, the child was older (14 years), there were no substantiated child related concerns and one substantiated parenting concern (1.042, S.E. = 0.238, *p* < 0.001).

The highest indirect effect for reported concerns about parental mental illness with moderators present, were in cases where the parent was not a single caregiver, the child age was older (14 years) and there were no other substantiated child concerns, but three substantiated parenting concerns (1.312, S.E. = 0.338, *p* < 0.001). The lowest indirect effect with moderators present were when there was a single caregiver, the child was older (14 years) there were no substantiated child related concern and one substantiated parenting concern (0.456, S.E. = 0.248, *p* = 0.066).

## Discussion

Our initial aim was to study who reports concerns about parental substance abuse or mental illness and what are the characteristics of these reports of concern. The results showed, not surprisingly, that a majority of these reports came from different healthcare services. Health care services refers to a large group of workers, such as public health nurses, school nurses, general practitioners, and hospital personnel. In their meetings with the patient, health personnel working with adult patients who struggle with mental health issues or substance abuse are in a key position to address the impact mental health problems may have on parenting quality. They are also an important agent to initiate collaboration with services such as the CWPS, which can provide supporting (and preventive) interventions for these families. However, we believe the reports of concerns from adult mental health services are still too low. Based on the large proportion of children and adolescents who have parents with mental illness or substance abuse in the general population, and on the large proportion of those who are in contact with the CWPS who have parents with such problems, it is reason to believe that the threshold to report concerns about COPMI is still too high. Even though COPMI have received a lot of attention in these services during the last decade, there still seems to be a long way to go before the collaboration between adult mental health services and the CWPS is adequate and leads to a functional service-provision (2).

A large proportion, about one third of the reports of concerns about PSA were reported from private parties, such as a relative, a friend or a neighbor. This is particularly disturbing if it means that non-mandated reporters of concern take on a larger responsibility for children at risk from neglect because of parental substance abuse, compared to professionals working with this group of patients/parents. These parents may not be identified as having such problems and may not receive help for their abuse problems from health care services. In such cases, reports of concern from private parties represent a safety net for children in families with substance abuse problems. The rest of the reported concerns related to such problems were reported by professionals that have a mandate to report. Because of the social stigma related to PSA, it is challenging for the CWPS to gather information about such problems through their contact with parents and children. In Norway, the police regularly report episodes of PSA to the CWPS, which can provide these services with important information and hence strengthen the potential for substantiation of concern and provision of services. From a prevention perspective, it is positive if parental mental illness and substance abuse is discovered while the children are relatively young, as interventions aimed at younger children have greater potential for strong effects on the child's development (43, 44).

Our second aim was to investigate direct and mediated effects of reports about COPMI and PSA upon service provision. We hypothesized that service provision is dependent upon the CWPS's investigation and subsequent substantiation of the reported problem. This hypothesis was confirmed. In terms of whether reports of concern were met with service provision by the CWPS, the results showed that total of 33.1% of reports with PSA and 41.7% of reports about parental mental illness were provided with services. Although the rate of service provision in cases where reports of concern is related to parental mental illness and/or substance abuse is similar to other types of reports, the analysis showed that there was a partial mediation effect that goes through the substantiation process. Primarily, this means that when parental mental health problems or substance abuse is reported, service provision is highly dependent upon the substantiation of such specific problems. Identification and substantiation of other types of problems seem less important. The CWPS's difficulties in substantiating parental mental illness and substance abuse emphasize the need for a broad assessment of child development, familial social resources and network, as well as general parenting problems in these cases (45).

It is an interesting finding that when the CWPS receive reports of concerns related to PSA, problems related to the development and health status of the child were less frequently identified. This may imply that PSA among parents is evaluated as important enough in itself, regardless of the child's developmental status. However, it may also imply that the CWPS become very focused on gathering “evidence” of substance abuse problems in the parents, as opposed to assessing the mental and social status and development of the child. If the latter is the case, the CWPS run the risk of being blind to the consequences of living with parental substance abuse, even though the services are finding it difficult to document such problems to a satisfactory degree. Havnen et al. (28) found that children placed out-of-home because of parental substance abuse had lower scores on mental health problems than children placed in out-of-home care because of other reasons. It is however possible that this is at least partly mediated by earlier intervention in cases with serious substance abuse, and that this protects the child from developing mental health problems later. Therefore, we would be very careful to dismiss the need for a broad assessment of the child's needs at the investigation phase of these cases.

When the reported concern was about parental mental illness, more parenting problems were identified. It is unclear why the CWPS are able to detect more parenting problems in these cases compared to cases where the concern is related to parental substance abuse. However, since it may be more intuitive why mental illness poses a threat to everyday parenting functions, it may seem more natural to assess such difficulties, as opposed to substance abuse problems which are difficult to substantiate by normal assessment procedures. It may be easier for parents to admit and seek help for mental health problems compared to substance abuse problems. Admitting to substance abuse problems in encounters with the CWPS may be considered more likely to invoke considerations of custody issues. Another explanation could be the stronger stigma connected to substance abuse problems than mental health issues, which in turn could make it easier for parents to admit and receive help for mental health problems.

Our final aim was to investigate if the thresholds for substantiation of parental mental health problems and parental substance abuse were moderated by other case characteristics. We hypothesized that the threshold for substantiation of cases was moderated by child age, single parent vs. two parent household and the number of other child and parenting problems that were identified. This hypothesis was rejected.

It is probable that the investigation process following reports about PSA in particular, more often aims to substantiate or unsubstantiate the substance use and that the investigation perhaps is not as broad and needs-oriented as in other cases. An implication of this may be that parents who struggle with substance abuse issues, may not be offered supporting interventions to prevent out-of-home placements. If this is the case one might question if this practice is in line with the broad family-oriented mandate of the Norwegian CWPS (33–35). However, it may also be the case that the CWPS have not implemented relevant interventions for these families. This interpretation is in line with the results of Christiansen et al. (31), who found that a lack of suitable interventions for these families hinders service-provision. As parental substance abuse represents a serious risk factor in child development, a lack of service provision in these cases needs to be met with implementation of adequate interventions and services by the CWPS.

It is also apparent that mental health problems are risk factors that the CWPS are not very likely to look for unless the concern has already been raised in the report. An implication of this may be that an important opportunity to discover COPMI is missed, because the CWPS does not systematically assess if the reported problems within a family may be due to mental illness or substance abuse. Subsequently, this has important implications for the partners of the CWPS that provide the reports of concerns, such as schools, health care services, social services, and the police. First, the reports should be specific and explicit about any suspicion that children are struggling due to parental mental health or substance abuse problems. If the report of concern is too vague or other vicarious concerns are provided as cause for the report to the CWPS, perhaps out of fear of offending parents or damaging a therapeutic relation, then the CPWS will most likely not look for such risk factors in their investigation. Although we do not know for certain how prevalent this problem may be, we do find it strange that private parties are so often the reporters of substance abuse and mental health problems. It is worrying if other public services are either not aware of or not sufficiently alarmed by such problems in the 25–30% of cases that are reported by family, friends, and neighbors. A solution to this could be to raise awareness among mandated reporters who work with children, e.g., schools, kindergartens, public health nurses, about the importance of identification and reporting of such problems.

Second, we do find it worrying that the CWPS assessment in cases with parental mental health problems or substance abuse seem to have a more limited scope than other reported concerns. This conclusion is supported by the failure in this study to identify any interaction effects between reported concern and child age, single care, number of child related problems or number of parenting problems on the substantiation decision. If the CWPS assessments had not primarily focused on the occurrence or non-occurrence of substance use and mental health problems but had also taken into consideration the effects this may have upon parents' ability to care for the child and on child functioning and health, such interaction effects should be expected. In a public health perspective, thresholds for substantiation of parental mental health problems and subsequent service provision should be much lower for young children of single caregivers due to the added effects of early intervention and prevention (3, 46). Additionally, with the current knowledge about the cumulative effects of family risk factors on child development and health (8), the threshold for substantiation and service provision should be lower when mal-adaption or child mental health problems are starting to manifest. Sadly, this does not seem to be the case since we found no evidence for the moderation effect hypothesis.

### Limitations

We do not have information about the types of substance use parents were reported for, or the types of substance use that was substantiated. We do however assume that certain types of substance abuse such as use of heroin or methamphetamines that may have a more easily identifiable effect upon users are more likely to be substantiated. Additionally, does different types of drugs have differing effects upon the user's ability to care for the child. It is therefore likely that the types of substances that are being used affect both the chance of detection, and the likelihood that it will be considered serious enough to constitute a problem.

We only included variables at case level in this study. As indicated in the introduction we do acknowledge that not all variation in child welfare decision making is determined by case level factors. It may be important which social worker is processing the case. How serious a concern has to be in order to be substantiated as risk is not only influenced by the facts of the case but also by how these facts are understood and interpreted by the decision-maker. Unfortunately, we are unable to control for variability in professional judgement because we do not have information about which workers were involved in which cases. We do know how cases are nested within agencies. However, because the study was designed primarily to look at case level factors, we have relatively few agencies included. Therefore, we consider analysis of variation at the agency level to be outside the scope of this article.

## Conclusions

The total effect of reports about mental illness and substance abuse upon service provision was low. This means that the overall chance that children and parents receive services from CWPS in these types of cases are about the same as when the reports of concern contain other types of problems. Service provision for reported concerns about substance abuse and/or mental illness is however much more dependent upon substantiation status. This indicates that action is likely taken in high-risk cases, but that preventive measures in low-risk cases are not as commonly provided.

## Data Availability Statement

The datasets presented in this article are not readily available because our license from the Data Protection Authority does not include permission to store raw data in open data repositories. Requests to access the datasets should be directed to svein.arild.vis@uit.no.

## Ethics Statement

The studies involving human participants were reviewed and approved by the Norwegian Centre for Research Data, the Council for Duty of Confidentiality and the Norwegian Data Protection Authority. Written informed consent from the participants' legal guardian/next of kin was not required to participate in this study following a decision by the directorate for children and family affairs.

## Author Contributions

SV designed the study. SV, KH, and CL collected the data. SV and BH conducted the analyses. All authors contributed to the writing of the article manuscript and agreed to be accountable for the content of the work.

## Funding

The study was funded by the Norwegian Directorate for Children, Youth and Family Affairs (Bufdir) and UiT The Arctic University of Norway.

The authors also want to thank the Norwegian Directorate for Children, Youth and Family affairs and UiT The Arctic University of Norway for funding the project. Additionally, the authors wish to thank the Research Support Team at RKBU/UiT The Arctic University of Norway.

## Conflict of Interest

The authors declare that the research was conducted in the absence of any commercial or financial relationships that could be construed as a potential conflict of interest.

## Publisher's Note

All claims expressed in this article are solely those of the authors and do not necessarily represent those of their affiliated organizations, or those of the publisher, the editors and the reviewers. Any product that may be evaluated in this article, or claim that may be made by its manufacturer, is not guaranteed or endorsed by the publisher.

## Abbreviations

COPMI: Children of parents with a mental illness
CWPS: Child Welfare and Protection Services
PSA: Parental Substance Abuse.
